# Bacillus cereus *cshA* Is Expressed during the Lag Phase of Growth and Serves as a Potential Marker of Early Adaptation to Low Temperature and pH

**DOI:** 10.1128/AEM.00486-19

**Published:** 2019-07-01

**Authors:** Marina Français, Frédéric Carlin, Véronique Broussolle, Christophe Nguyen-Thé

**Affiliations:** a UMR408 SQPOV Sécurité et Qualité des Produits d’Origine Végétale, INRA, Avignon, France; b UMR408 SQPOV Sécurité et Qualité des Produits d’Origine Végétale, Avignon Université, Avignon, France; Rutgers, The State University of New Jersey

**Keywords:** *Bacillus cereus*, RNA helicase, *abrB*, acid adaptation, cold adaptation, *cshA*, lag phase

## Abstract

Bacterial adaptation is characterized by a lag phase during which cells do not multiply or modify their physiology to cope with the constraints of their environment. Our aim was to determine a sequence of events during the lag phase of growth at low temperature and pH for three Bacillus cereus strains. The onsets of expression of two genes, one of which is essential for stress adaptation (*cshA*, coding for a RNA helicase) and one of which is involved in the transition between lag phase and exponential phase (*abrB*, coding for a transition regulator), were determined using fluorescent transcriptional reporter systems. Regardless of the stressing conditions and the tested strains, the *cshA* promoter was active very early, while the biomass increased and always did so before the first cell division. At 12°C and pH 7.0, the onset of *cshA* promoter activity occurred at between 3 h and 7 h, while the bacterial counts started to increase at between 12 h and 13 h. At pH 5.0 and at 20°C or 30°C, the onset of *cshA* promoter activity occurred before 1 h and earlier than at pH 7.0. In contrast, the onset of *abrB* promoter activity depended on the strain and the stressing conditions. In the ATCC 14579 strain, the onset of *abrB* promoter activity always started at between 30 min and 3 h, before biomass increased and cell division occurred. For the other strains, it took place along with the first cell division at 12°C but did so much later during growth under the other tested conditions.

**IMPORTANCE** The spore-forming bacterium B. cereus is a major cause of foodborne outbreaks in Europe. Some B. cereus strains can grow at low temperatures and low pH in many processed foods. Modeling of the bacterial lag time is hampered by a lack of knowledge of the timing of events occurring during this phase. In this context, the identification of lag phase markers, not currently available, could be a real advance for the better prediction of lag time duration. Currently, no molecular markers of this phase are available. By determining that *cshA* was always expressed early during the lag phase, we provide a molecular marker of the early adaptation process of B. cereus cells when exposed to low temperature and pH.

## Abstract (precis)

The spore-forming bacterium B. cereus is a major cause of foodborne outbreaks in Europe. Some B. cereus strains can grow at low temperatures and low pH in many processed foods. Modeling of the bacterial lag time is hampered by a lack of knowledge of the timing of events occurring during this phase. In this context, the identification of lag phase markers, not currently available, could be a real advance for the better prediction of lag time duration. Currently, no molecular markers of this phase are available. By determining that *cshA* was always expressed early during the lag phase, we provide a molecular marker of the early adaptation process of B. cereus cells when exposed to low temperature and pH.

## INTRODUCTION

Bacillus cereus sensu lato, a major cause of foodborne outbreaks in Europe (1), comprises several closely related species associated with diarrheal and emetic types of foodborne illness (2). The diarrheal illness is caused by enterotoxins produced in the gastrointestinal tract of the host, whereas the emetic illness is caused by cereulide, a toxin produced in foods. The species B. cereus sensu stricto, B. thuringiensis, B. cytotoxicus, B. weihenstephanensis, B. wiedmannii, and B. toyonensis, all included in B. cereus sensu lato (3), produce some of these toxins. B. cereus sensu lato forms heat-resistant spores that may survive, germinate, and grow during the distribution or storage of foods, even under cold conditions. B. cereus sensu lato exhibits a wide domain of growth temperatures and pHs. Some strains are psychrotrophic, while others are moderately thermophilic, and growth at pH 4.3 has been reported for some of them (4). B. cereus sensu lato may therefore colonize foods in diverse thermal environments and adapt to the diversity of pHs created by food ingredients. Psychrotrophic strains can grow at temperatures ranging from 4 to 5°C, which, in association with the ability of their spores to survive pasteurization treatments (5), makes them an important hazard for heat-treated and refrigerated foods. Besides, inappropriate consumer practices regarding the cooling and storage of foods (6) also allow the multiplication of mesophilic strains of B. cereus sensu lato.

To cope with low temperatures and low pH, bacteria implement adaptive solutions, implying various molecular and physiological mechanisms (7–9). At low temperatures, for example, B. cereus modifies glucose metabolism (10) and membrane fatty acid composition (11, 12); overexpresses specific proteins, such as DNA gyrases, cold acclimation proteins (CAPs), and cold shock proteins (CSPs) (13); or activates two-component systems, such as CasKR, essential for B. cereus growth at low temperature (14). We have specifically shown that the expression of RNA helicase-encoding genes is also a major determinant of B. cereus ATCC 14579 cold adaptation (15). The translation process depends on the mRNA conformation and is impaired or prevented by secondary mRNA structures induced during growth at low temperature. In response, the RNA helicases of B. cereus, B. subtilis, Listeria monocytogenes, or Escherichia coli, for instance, unroll detrimental secondary structures impairing growth (16–19). In B. cereus ATCC 14579, the RNA helicase-encoding genes *cshA*, *cshB*, and *cshC* are upregulated and required for growth in response to low temperature. Deletion of each of these genes, in particular, *cshA*, prevents or impairs growth and changes the morphology of cells growing at low temperature (18). Besides, a *cshA* deletion in the B. cereus ATCC 14579 strain extended the growth lag time at pH 5.0 compared to pH 7.0 (20).

Bacteria initiate growth under suboptimal conditions by a latency or lag phase without any cell multiplication, during which a physiological adaptation takes place. The lag phase duration (lag time) increases as the temperature decreases; for instance, it is increased at 12°C compared to 30°C (14, 20). The lag phase also increases when the pH strays from the optimum or after exposure to other physical or chemical stresses (21, 22). The lag time of natural bacterial contaminants in foods is poorly predictable (23), causing uncertainty in the assessment of pathogenic bacteria, such as B. cereus, in foods. The end of the lag phase is usually defined as the time at the onset of cell division (measured by the increase in CFU counts, for instance) or as the time at the onset of biomass increase (usually appraised by the increase in culture turbidity) (21). However, bacterial cells may initiate physiological activity and gene expression for adaptation to changing nutrient and environmental conditions before any cell division and increase in biomass (24). Some genes are expressed during the lag phase; for instance, *abrB* gene expression increases in the transition of B. subtilis and B. cereus cells from quiescence to growth (25–27).

Our objective was to improve our knowledge of the sequence of events occurring during B. cereus sensu lato lag phase and early growth at low temperature and/or low pH. We determined the onset of expression of the *cshA* and *abrB* genes, necessary for *Bacillus* cold and low-pH adaptation (*cshA*) and involved in the transition between quiescent and actively growing cells (*abrB*). We followed the activity of these gene promoters over time, measured by using fluorescent transcriptional reporter systems monitored by spectrofluorimetry, concomitantly with changes in biomass and cell division, measured by determining the absorbance and CFU counts, respectively. Two mesophilic strains (strains ATCC 14579 and ATCC 10876) and one psychrotrophic strain (strain MM3) were studied.

## RESULTS

### Monitoring *cshA* promoter activity during Bacillus cereus growth.

**FIG 1:**
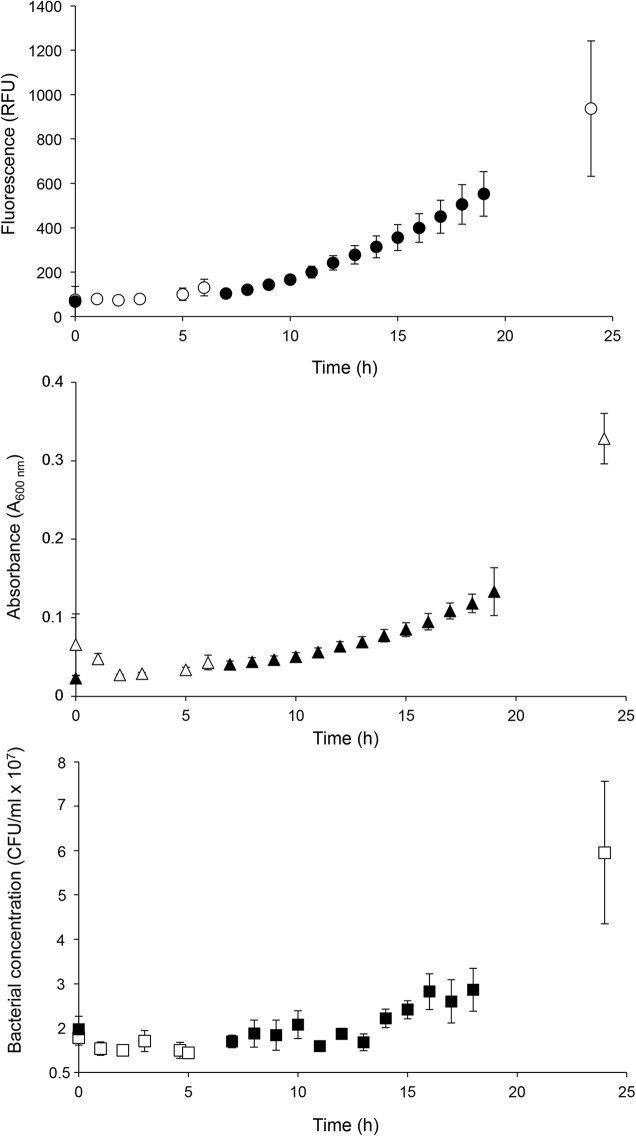
Fluorescence monitoring (relative fluorescence units [RFU]) (•), determination of the *A*_600_ (▲), and determination of the bacterial counts (number of CFU per milliliter) (■) of a culture of the B. cereus ATCC 10876 strain harboring the P*cshA*′*gfp* transcriptional fusion in mAOAC broth at 12°C and pH 7.0 (values are the mean ± SD; *n* = 3). Closed and open symbols represent the results of experiments with different sampling times.

Changes over time in the *cshA* promoter activity (followed by determination of the fluorescence of the green fluorescent protein [GFP]), biomass (represented by *A*_600_), and cell division (represented by the number of CFU per milliliter) of B. cereus strain ATCC 10876-P*cshA′gfp*, as an example, grown at 12°C and pH 7.0 are presented in Fig. 1. In some cases, as can be observed in Fig. 1, we observed different phases in fluorescence: first, a stable level of fluorescence; second, an increase concomitant with that of the *A*_600_ but before the increase in the number of CFU per milliliter; and third, a continuation of the increase in fluorescence during growth. In other cases (data not shown), the second phase was not observed and the increase in fluorescence occurred concomitantly with or after population growth. The onsets of the increase of these three variables, the onset of the fluorescence increase (λ*_F_*), the onset of the *A*_600_ increase (λ*_A_*), and the onset of increase in the number of CFU per milliliter (λ*_C_*)_,_ respectively, were estimated for each individual growth experiment by the intersection points between the horizontal and the increasing parts of the curve (as described in Materials and Methods).

**FIG 2:**
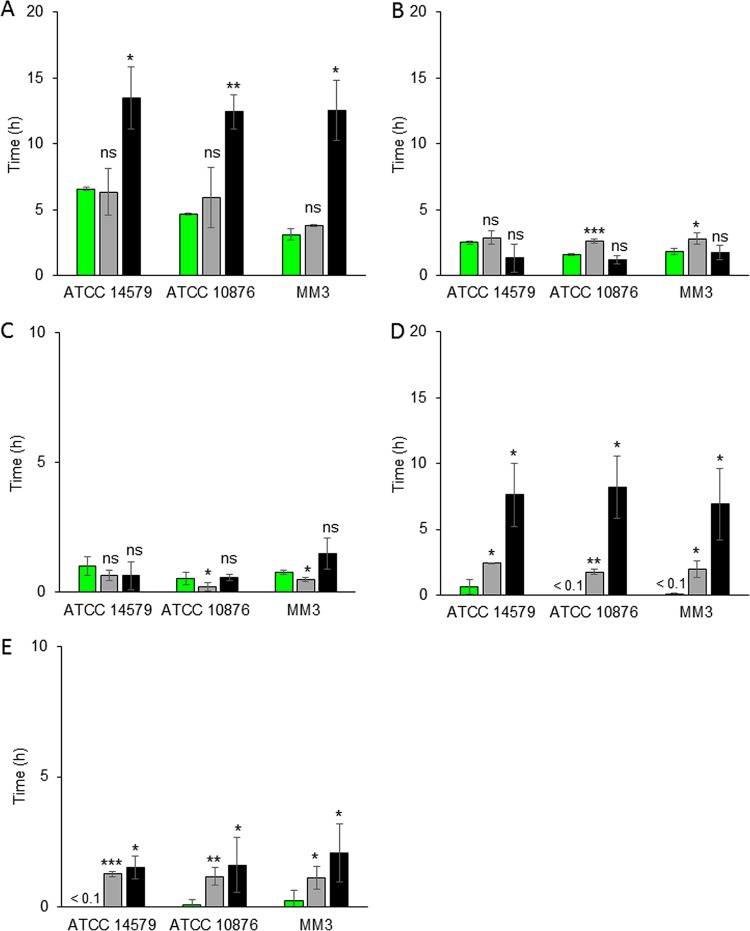
Times of onset of *cshA* promoter activity (λ*_F_*) (green bars), an *A*_600_ increase (λ*_A_*) (gray bars), and a plate count increase (λ*_C_*) (black bars) (values are the mean ± SD; *n* = 3). Experiments were done at 12°C (A), 20°C (B and D), or 30°C (C and E) and at pH 7.0 (A, B, and C) or pH 5.0 (D and E) for B. cereus sensu lato ATCC 14579, ATCC 10876, and MM3. The notation <0.1 indicates that λ*_F_* is shorter than 0.1 h. Asterisks above the bars show significant differences (determined by Student's *t* test) between λ*_F_* and λ*_A_* or λ*_C_*. *, *P* < 0.05; **, *P* < 0.01; ***, *P* < 0.001; ns, no significant difference.

λ*_F_*, λ*_A_*, and λ*_C_* were measured for the three tested B. cereus sensu lato strains grown at 12°C, 20°C, and 30°C and in mAOAC (which is made of synthetic AOAC broth [HiMedia Laboratories]) at pHs 7.0 and 5.0. Conditions of pH 5.0 and 12°C were not tested, as a previous study reported that B. cereus does not grow under such conditions (28). For all strains, the onset of P*cshA* activity (λ*_F_*) occurred at 12°C and pH 7.0 at between 3.3 h and 6.6 h, without any observable increase in CFU counts (Fig. 2A). Independently of the studied strain, λ*_C_* values were between 12.5 h and 13.5 h, whereas λ*_A_* values were between 3.8 h and 6.3 h and shorter than λ*_C_*. Under cold conditions, bacterial cells tend to elongate, which may explain the *A*_600_ increase observed during cold adaptation several hours before the increase in CFU counts (29). An increase in the *A*_600_ has already been observed during the lag phase (i.e., before cell division) of B. cereus under optimal and low-pH conditions (30), and cell elongation before cell division at a cold temperature was previously observed (31). No significant differences between λ*_A_* and λ*_F_* were verified. In other words, the onset of the biomass increase of the three tested B. cereus strains occurred at the same time as the onset of P*cshA* activity. At 12°C and pH 7.0, *cshA* was expressed early during cold adaptation, at the same time as cell elongation and before cell division.

Lag times were shorter, unsurprisingly, when the incubation temperature was increased from 12°C to 20°C or 30°C at pH 7.0. λ*_C_* was between 1.2 h and 1.7 h at 20°C (Fig. 2B) and between 0.5 h and 1.5 h at 30°C (Fig. 2C). λ*_A_* was between 2.6 h and 2.9 h at 20°C (Fig. 2B) and between 0.2 h and 0.6 h at 30°C (Fig. 2C). In the ATCC 14579 strain, no significant differences were observed between λ*_A_*, λ*_C_*, and λ*_F_*, and thus, P*cshA* activity appeared concomitantly with the beginning of cell division and the increase in the *A*_600_. In the ATCC 10876 and MM3 strains, λ*_F_* and λ*_C_* were not significantly different, but they were significantly different from λ*_A_*, and P*cshA* was active at the same time as the first cell division.

When the pH of the medium was lowered to 5.0, λ*_C_* values were extended by approximately 1 h at 30°C (Fig. 2E) and by 7 to 8 h at 20°C compared to those at pH 7.0 (Fig. 2D). This longer lag phase of the three tested strains, markedly longer at pH 5.0 than at pH 7.0, suggests physiological stress induced by increased acidity, an aspect already evidenced for B. cereus (32). At 20°C, λ*_A_* occurred at 2 h (Fig. 2D) and at 1.2 h at 30°C (Fig. 2E). At 20°C and pH 5.0, λ*_A_* values were similar to those at 20°C and pH 7.0, and consequently, the difference between λ*_C_* and λ*_A_* increased when the pH decreased. At pH 5.0, λ*_A_* was shorter than λ*_C_*, indicating cell elongation before cell division, as observed at 12°C. While λ*_C_* was markedly longer at pH 5.0 than at pH 7.0 for all strains, the λ*_F_* of cells of the three tested strains exposed to pH 5.0 and incubated at both temperatures (20°C and 30°C) was shorter than 1 h, i.e., much shorter than that at pH 7.0. This suggests that P*cshA* activity started earlier when cells were grown at pH 5.0 than at pH 7.0.

**FIG 3:**
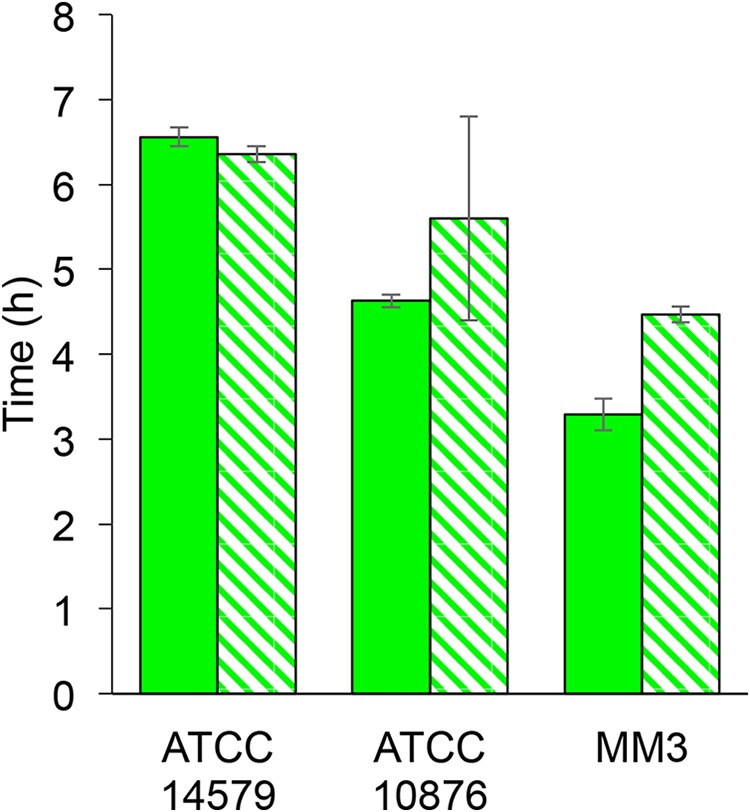
Times of onset of *cshA* promoter activity for the B. cereus ATCC 14579, ATCC 10876, and MM3 strains with a starting inoculum made of vegetative cells (full bars) or spores (hatched bars) (values are the mean ± SD; *n* = 2 for spores and *n* = 3 for vegetative cells).

We also investigated the activity of P*cshA* at 12°C and pH 7.0 in a bacterial culture initiated with a spore inoculum. Growth, i.e., the increase in *A*_600_ and the number of CFU, from the spore inoculum was similar to that from the vegetative cell inoculum. The only difference was a decrease in the *A*_600_ within the first minutes of incubation when spores germinate, as previously described (33). This germination phase was excluded to determine the time of onset for the *A*_600_ increase. At 12°C and pH 7.0 with an inoculum of spores, λ*_F_* values were between 4.5 h and 6.3 h, depending on the strain, and were close to those of vegetative cells, which were between 3.3 h and 6.5 h (Fig. 3). Regardless of the inoculum (spores or vegetative cells), P*cshA* activity onset occurred before the increase in the number of CFU.

### Monitoring *abrB* promoter activity during Bacillus cereus growth.

**FIG 4:**
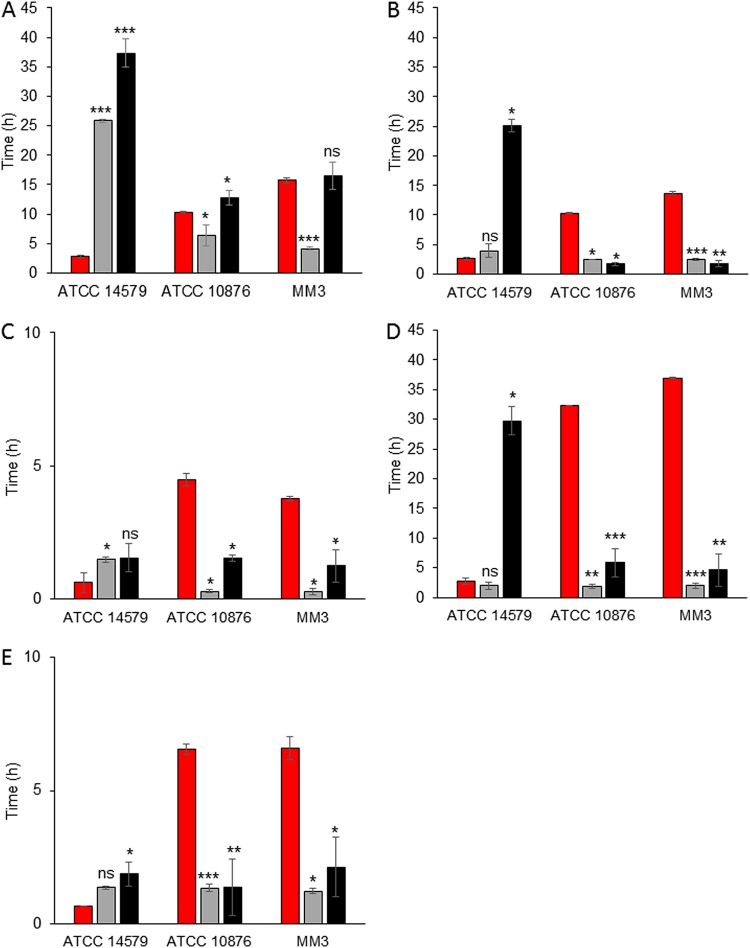
Times of onset of *abrB* promoter activity (λ*_F_*) (red bars), an *A*_600_ increase (λ*_A_*) (gray bars), and a plate count increase (λ*_C_*) (black bars) (values are the mean ± SD; *n* = 3). Experiments were done at 12°C (A), 20°C (B and D), or 30°C (C and E) and at pH 7.0 (A, B, and C) or pH 5.0 (D and E) for the B. cereus sensu lato ATCC 14579, ATCC 10876, and MM3 strains. Asterisks above the bars show significant differences (determined by Student's *t* test) between λ*_F_* and λ*_A_* or λ*_C_*. *, *P* < 0.05; **, *P* < 0.01; ***, *P* < 0.001; ns, no significant difference.

In the ATCC 10876 and MM3 strains, at 12°C, λ*_F_* was between 10.3 h and 15.7 h, which was significantly shorter or similar to that of λ*_C_* (which was between 12.8 h and 16.5 h). It marked the first cell division but was longer in all instances than λ*_A_* (which was between 4.1 h and 6.4 h), marking the onset of the biomass increase (Fig. 4A). In contrast, there was an early onset of *abrB* promoter activity (P*abrB*) in B. cereus strain ATCC 14579 (λ*_F_* = 2.8 h), which was much earlier than λ*_A_* (25.9 h) and λ*_C_* (37.3 h) (Fig. 4A). In other words, P*abrB* activity in B. cereus strain ATCC 14579 started long before the increase in biomass and cell division.

At 20°C and pH 7.0 in the B. cereus ATCC 10876 and MM3 strains, P*abrB* activity started at 10.3 h and 13.6 h, respectively, and after the first cell division and biomass changes (Fig. 4B). Although the differences between λ*_F_*, λ*_C_*, and λ*_A_* were lower at 30°C than at 20°C, P*abrB* activity still started significantly longer after biomass changes and the first cell divisions (Fig. 4C). In these two strains and under these conditions, the *abrB* gene was not expressed during the early stages of cell growth kinetics. In contrast, for ATCC 14579, λ*_F_* varied from 2.7 h at 20°C and pH 7.0 (Fig. 4B) to 0.6 h at 30°C and pH 7.0 (Fig. 4C), just before or at the same time as the *A*_600_ increase. At 30°C, it also occurred with the first cell divisions. In this strain, P*abrB* appeared to be active before or at the same time as biomass modifications and cell divisions, depending on the temperature. During the extended lag phase caused by exposure to pH 5.0, P*abrB* was active after the onset of cell division in ATCC 10876 and MM3 (λ*_F_* = 6.5 h and 6.6 h, respectively) but was active much earlier and before the onset of cell division in ATCC 14579 (λ*_F_* = 0.6 h) (Fig. 4D and E).

The time sequence of *abrB* expression would therefore be compatible with a role in the transition from lag to exponential phase when B. cereus is growing at 12°C. At a higher temperature or a low pH, its expression occurred too late in strains ATCC 10876 and MM3, suggesting an actual role in the transition between lag and log phases.

The growth of ATCC 14579-P*abrB′mCherry* at 20°C and 12°C was markedly delayed compared to that of ATCC 10876-P*abrB′mCherry* and MM3-P*abrB′mCherry* (Fig. 4A, B, and D). In addition, the lag time of ATCC 14579-P*abrB′mCherry* was longer and its growth rate was lower than those for the parental strain, whereas the growth of all other derivative strains was similar to that of the corresponding wild-type strain (data not shown). Our assumption is that the large quantity of the mCherry protein produced as a result of P*abrB* activity mobilized massive energy resources from the cell, which could explain the observed delay in growth. Nevertheless, this slowed growth pattern did not change the early expression of the *abrB* gene in strain ATCC 14579-P*abrB′mCherry*.

### GFP, mCherry stability, and microscopic observations.

**FIG 5:**
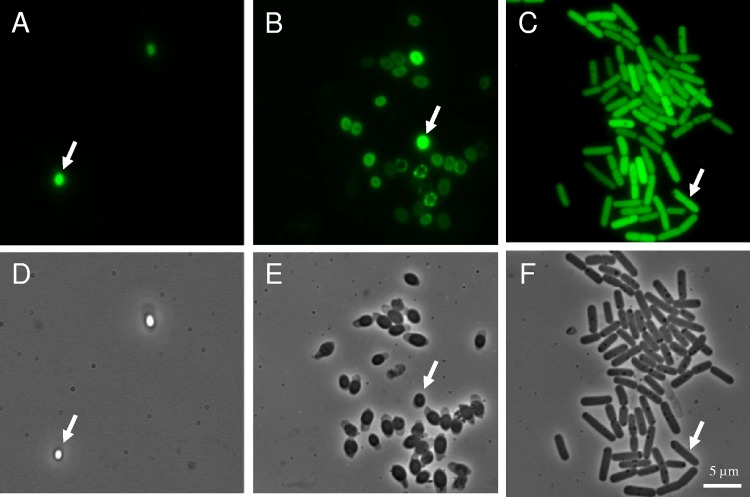
Fluorescence (A, B, C) and phase-contrast (D, E, F) images of the B. cereus ATCC 10876 strain harboring a P*cshA*′*gfp* transcriptional fusion in spores (A and D), germinated spores (B and E), and vegetative cells (C and F). Arrows show a typical spore (A and D), germinated spore (B and E), and vegetative cell (C and F). Magnifications, ×1,000.

**FIG 6:**
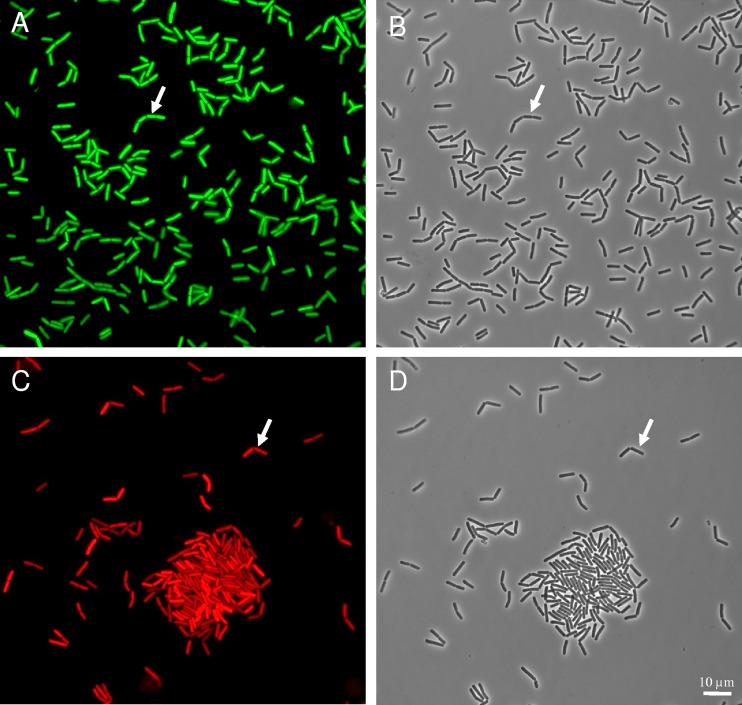
Fluorescence (A and C) and phase-contrast (B and D) images of the B. cereus ATCC 14579 strain harboring the P*cshA*′*gfp* (A, B) and P*abrB*′*mCherry* (C, D) fusions. Cells grown at 12°C for 8 h in mAOAC broth were concentrated by a gentle centrifugation and observed with an epifluorescence microscope. Arrows show the same cell in phase-contrast and in fluorescence images. Magnifications, ×1,000.

We showed that the half-life of the GFP and mCherry fluorescence in the transformed cells was longer than 100 h and, therefore, longer than the incubation times. To achieve an optical density (OD) of 0.5 at 30°C, inocula were harvested at between 3 and 4 h, at or after the onset of GFP and mCherry production. Therefore, cells were already fluorescent at the start of the experiment, as illustrated in Fig. 1. In the case of P*cshA* construction, the spores were themselves fluorescent (Fig. 5). Spores are metabolically inactive, and so is P*cshA* in spores, presumably. Considering the stability of GFP, spore fluorescence was likely due to the previous accumulation of GFP before or during spore formation. During the duration of the growth experiments, all cells were fluorescent, as illustrated in Fig. 6. Therefore, microscopic observation did not allow visualization of an on-versus-off state of promoter activity. The quantitative measure of fluorescence at the population level with the spectrofluorimeter was more appropriate to detect the increase in *cshA* and *abrB* promoter activity. However, microscopic observation showed that under all the tested conditions, *cshA* and *abrB* were expressed in all cells.

## DISCUSSION

Our work presents the time sequence of *cshA* and *abrB* promoter activity, biomass increase, and cell division during the early growth phase of three B. cereus sensu lato strains, two mesophilic strains (ATCC 14579 and ATCC 10876) and a psychrotrophic strain (MM3). It should be kept in mind that all the lag times obtained during these experiments are specific to our inoculation conditions and were just above the detection limit of the Tecan apparatus. The number of bacteria in the culture influences the lag time (34), and a lower cell concentration in the inoculum would presumably increase the lag time. The lag time is also dependent on the history of the cells constituting the inoculum. When cells are preadapted to stressful conditions, they have a shorter lag time than nonpreadapted ones (35, 36). In our study, cells were not preadapted, as our purpose was to study the phenomena occurring during adaptation, and the inoculum was produced at 30°C and pH 7.0, which presumably extended the lag time compared to that for preadapted cells.

The *cshA* gene encodes a DEAD box RNA helicase. RNA helicases are involved in diverse mechanisms, such as the unwinding of secondary structures for RNA degradation or the rearranging of RNP complexes during ribosome biogenesis, although in some bacterial species their molecular function could not be defined precisely (37, 38). We described, in previous studies, the involvement of *cshA* in B. cereus cold adaptation (18) and its higher expression in the early growth phase than in the late exponential or stationary phase (15). In this study, thanks to the very frequent monitoring of P*cshA* activity using a fluorescent reporter system, we showed that it was active either before or simultaneously with the initiation of cell division and biomass increase, whatever incubation temperatures (30°C, 20°C, and 12°C) and pHs (7.0 and 5.0) were used.

Under optimal growth conditions (30°C and pH 7.0), the *cshA* promoter was active concomitantly with the onset of growth. This is consistent with the previous observation that the growth of the B. cereus ATCC 14579 Δ*cshA* strain was impaired under nonstressful conditions, although to a much lesser extent than under stressful conditions (18). At a low pH and/or low temperatures, *cshA* was expressed at an earlier stage than under nonstressing conditions, several hours before the onset of cell division, suggesting a crucial role in adaptation to these difficult environments. At a low temperature, P*cshA* activity occurred before cell division but concomitantly with the onset of biomass increase. This is compatible with the role of CshA in the initiation of cell elongation and cell division. At low pH, P*cshA* activity occurred rapidly after inoculation in acid medium and a few hours before any biomass increase. This suggests that CshA may initiate some processes necessary for cell elongation and cell division during the adaptation of B. cereus to acid conditions. A previous study showed that a B. cereus mutant in which *cshA* was deleted had an altered growth at low pH (20). The sole RNA helicase, RhpA, of the gastric pathogen Helicobacter pylori, which is a homolog to CshA, was recently shown to be necessary for the adaptation and growth of this bacterium in the stomach of mice (39). However, the mechanisms underlying the role of RNA helicases in acid adaptation are still not known. In contrast, their involvement in cold adaptation has been described for several species (16–19). A DEAD box RNA helicase, such as CshA, could be implicated in the unwinding of the secondary structures of mRNA for optimized protein synthesis, mRNA degradation via the degradosome complex, or the rearrangement of RNA-protein (RNP) complexes during ribosome biogenesis (37, 38). At a low temperature, a huge decrease in the translational process is observed in bacterial cells, probably due to the presence of stabilized secondary structures in mRNA. To overcome this problem, cells synthesize RNA helicases to resolve and prevent the misfolding of RNA molecules by unwinding the unfavorable secondary structures, leading to single-stranded RNA. The early expression of *cshA* under cold conditions would be consistent with its role in the adaptation of translation (38). The mesophilic and psychrotrophic strains that we tested exhibited a similar early activity of their *cshA* promoter, suggesting that *cshA* may have a similar importance in adaptation to temperature and pH during lag phase among B. cereus sensu lato strains. In any case, the early activation of *cshA* under both low-temperature and low-pH conditions in all studied strains could be exploited as a marker of the onset of adaptation activity in B. cereus, before the end of the lag time, defined as the first detectable increase in cell numbers.

The *abrB* gene codes for an important regulator in *Bacillus* spp. (25) which is involved in various mechanisms, such as the transition from growth to stationary phase (40), sporulation (41, 42), biofilm formation (43), antibiotic synthesis (44, 45), or cereulide production in B. cereus (26). The *abrB* gene was also selected in our study for its involvement as a regulator in the transition from the lag phase to exponential phase (25). Although they are not strictly homologs, the nucleoid binding protein AbrB (found in B. subtilis) and Fis (from E. coli and Salmonella enterica serovar Typhimurium) display some similarities in size, DNA-binding characteristics (each contains one helix-turn-helix motif, located in the C terminus of the protein), growth cycle-dependent patterns of expression, and control over the expression of a range of operons (25). Fis is involved in the end of the lag phase (46). The accumulation in B. subtilis of *abrB* mRNAs during lag phase was maximal, but *abrB* mRNAs became undetectable before the mid-exponential phase (25). B. cereus genomes contain several *abrB* genes, and the one that we selected has the highest sequence and position homology with the *abrB* gene expressed early in another *Bacillus* sp. (25, 26). Under our tested conditions, the onsets of *abrB* gene expression were different among the tested strains. In B. cereus strains ATCC 10876 and MM3 at 12°C, *abrB* expression onset occurred as lag phase ended, i.e., at the first cell division, while at 20°C and 30°C, *abrB* expression was delayed. At 12°C, the bacterium placed under stressing conditions might have set up specific adaptation mechanisms involving *abrB*. P*abrB* activity was detected later in cells exposed to low pH than it was in cells exposed to pH 7.0 and well after growth initiation. In these strains, *abrB* did not seem to be involved in adaptation to acid stress. P*abrB* was active earlier in the ATCC 14579 strain than in the two other strains, either before or simultaneously with the entry of the cells into exponential growth phase. The role and the importance of *abrB* in stress adaptation and the timing of expression during lag phase in B. cereus sensu lato may therefore be strongly strain dependent. The expression level of the *abrB* gene during growth until mid-exponential phase in LB medium at 30°C was different in the B. cereus ATCC 14579 and ATCC 10987 strains (47), but this does not explain the marked differences in the timing of P*abrB* activation among strains and growth conditions.

The relationship between growth rates and temperature, pH, and other factors is now reasonably well established for foodborne pathogens, and growth simulations under a wide range of conditions are now accessible using online tools (48). In contrast, the modeling of a bacterial lag time is still unsatisfactory in the particular absence of a precise determination of when the various adaptation mechanisms occur. Besides, bacterial adaptation can interfere with the strategies of agrifood industries aiming at mild processing and preservation to control the growth of pathogenic bacteria while maintaining the organoleptic and nutritional qualities of food. In this context, the identification of the stages and physiological states of growing microorganisms through the use of molecular markers could be a real advance in the prediction of the lag time duration. Currently, biochemical, morphological, physiological, or transcriptional markers exist, but no molecular markers of lag phase are available (24). Gene expression is one of the fastest responses of bacteria facing a new environment and has previously been used to define molecular biomarkers of the physiological state of B. cereus (49–54). Gene expression was also used to characterize the phases of cellular differentiation (55, 56). Since the growth of B. cereus in food can lead to foodborne illness or food spoilage (57), causing huge financial costs for agrifood industries, the monitoring of *cshA* expression could provide a rapid way to determine when bacterial cells are ready to start growing without making cell counts. The absence of gene expression would indicate that cells have still not emerged from the lag phase. At low temperatures, *cshA* expression corresponds perfectly to the lag phase output, while it occurs earlier at low pH, making *cshA* even safer as a lag phase marker. In our work, to identify genes that are potential lag phase markers, we used a fluorescent reporter system, which allowed the combination of the batch monitoring of expression with the microscopic observation of each cell. In challenge testing, currently used in the food industry to verify the safety of foods, it would be possible to spike food matrices with B. cereus strains carrying the *cshA* promoter coupled with luminescent reporters, as previously reported to detect B. cereus toxin production in products (58). Other techniques could be used to detect *cshA* expression in food samples naturally contaminated with low numbers of B. cereus bacteria, such as the droplet digital reverse transcription-PCR. This technique was recently used to target B. cereus in a food sample (milk) (59). Information on the lag time and adaptation provided by *cshA* expression could be combined with predictive microbiology models to better integrate the specific stress behavior of foodborne pathogenic bacteria and improve food processes, as proposed by Havelaar et al. (60).

Although the discovery of potential new biomarkers allows new perspectives, these new data must first be integrated into mathematical models that predict bacterial growth under the conditions found in the food industry. In conclusion, the *cshA* gene could potentially be a reliable molecular marker for the early adaptation of B. cereus exposed to the low temperatures and the pHs prevalent in foods and in the food chain because of its systematic expression during lag phase, before the initiation of growth, demonstrated for three B. cereus strains under our tested conditions.

## MATERIALS AND METHODS

### Bacterial strains and growth conditions.

Bacillus cereus strains ATCC 14579 and ATCC 10876 (both mesophilic strains able to grow at between 10°C and 45°C) and strain MM3 (a psychrotrophic strain able to grow at between 7°C and 40°C and recently affiliated with B. wiedmannii [61]) were the parental strains of the recombinant fluorescent derivatives (Table 1) used in the experimental work. Escherichia coli DH5α (62) was used as the host strain for plasmid construction. An unmethylated plasmid was prepared with E. coli SCS110 (63) before transfer into B. cereus by electroporation. Strains were stored at −80°C in a 30% glycerol solution. E. coli cultures and routine cultures of B. cereus were carried out in Luria-Bertani (LB) broth or agar (Biokar). Transformed B. cereus and E. coli strains were selected on LB agar supplemented with erythromycin to a final concentration of 5 μg/ml or ampicillin to a final concentration of 100 μg/ml, respectively. For experimental purposes, B. cereus cells were grown in mAOAC (which is made of synthetic AOAC broth [Wright and Mundy M334 broth; HiMedia Laboratories]) at pH 7.0, sterilized by autoclaving at 121°C for 20 min, and supplemented with a filter-sterilized glucose solution to a final concentration of 6.9 mM. When necessary, the pH of mAOAC was adjusted to 5.0 by the addition of 1 M HCl. mAOAC was previously used to study B. cereus cold adaptation (64) and exhibits a low self-fluorescence under the tested conditions. Cultures were incubated at 12°C, 20°C, and 30°C in a temperature-controlled incubator. B. cereus spores were produced as previously described (63), counted, and stored in cold demineralized water at −20°C for up to 6 months.

**TABLE 1:** Plasmids and strains used in this study

Plasmid or strain	Description	Reference or source
Plasmids		
pHT315-*gfp*	Contains the *gfp-mut1* coding sequence between the XbaI and HindIII restriction sites of plasmid pHT315	66
pHT304-18Ω*mCherry-gfp*	Contains a ribosome binding site and *mCherry* (optimized for *Bacillus* spp.) and *gfp* coding sequences	Gift from M. Gohar and L. Slamti
pHT315-P*cshA*′*gfp*	Fluorescent reporter plasmid construct for *cshA* transcriptional activity	This study
pHT304-18ΩP*abrB*′*mCherry-gfp*	Fluorescent reporter plasmid construct for *abrB* transcriptional activity	This study
B. cereus strains		
ATCC 14579	Wild type	67
ATCC 10876	Wild type	68
MM3	Wild type	69
ATCC 14579-P*cshA*′*gfp*	ATCC 14579 carrying pHT315-P*cshA*′*gfp*	This study
ATCC 10876-P*cshA*′*gfp*	ATCC 10876 carrying pHT315-P*cshA*′*gfp*	This study
MM3-P*cshA*′*gfp*	MM3 carrying pHT315-P*cshA*′*gfp*	This study
ATCC 14579-P*abrB*′*mCherry*	ATCC 14579 carrying pHT304-18ΩP*abrB*′*mCherry*	This study
ATCC 10876-P*abrB*′*mCherry*	ATCC 10876 carrying pHT304-18ΩP*abrB*′*mCherry*	This study
MM3-P*abrB*′*mCherry*	MM3 carrying pHT304-18ΩP*abrB*′*mCherry*	This study

### Construction of the fluorescent reporter transcriptional fusions.

We fused the promoter region of the *cshA* (BC0259 in ATCC 14579, bcere0002_2050 in ATCC 10876, and bcere0006_2150 in MM3) and *abrB* (BC0042 in ATCC 14579, bcere0002_260 in ATCC 10876, and bcere0006_260 in MM3) genes with reporter genes coding for fluorescent proteins. The *cshA* gene promoter was associated with the *gfp* gene reporter, and the *abrB* gene promoter was associated with a B. thuringiensis-optimized *mCherry* gene (55).

The DNA sequence containing the *cshA* promoter was amplified from genomic DNA by PCR using primers P*cshA*-Fw-*EcoR*I/P*cshA*-Rv-BamHI and inserted into pHT315-*gfp* to generate pHT315ΩP*cshA′gfp* (Table 1). The DNA sequence containing the *abrB* promoter was amplified from genomic DNA by PCR using primers P*abrB*-Fw-SphI/P*abrB*-Rv-XbaI (Table 2) and inserted into pHT304-18Ω*mCherry-gfp* to generate pHT304-18ΩP*abrB*′*mCherry-gfp* (Table 1). Oligonucleotide primers were synthesized by Eurogentec (Table 2).

**TABLE 2:** Primer sequences^*a*^

Primer	Sequence	*T_m_* (°C)
P*cshA*-Fw-EcoRI	GCAGGAATTCCAAATTGCTGAAGGAGCAAA	55.2
P*cshA*-Rv-BamHI	TGCTGGATCCAAAAGGCGTTTTCCGAATTT	55.2
P*abrB*-Fw-SphI	GCGCATGCTCGTATAAGCATGTCCAATG	56.3
P*abrB*-Rv-XbaI	CGCGTCTAGAAGATTTCAAGAGCGTCCTTT	56.5

a Restriction sites are underlined. *T_m_*, melting temperature.

Plasmid DNA was extracted from B. cereus and E. coli by a standard alkaline lysis procedure using a Wizard SV miniprep purification system (Promega), with an additional incubation at 37°C for 1 h with 5 mg of lysozyme for the lysis of B. cereus cells. Chromosomal DNA was extracted from B. cereus cells harvested during the mid-log phase. Restriction enzymes and T4 DNA ligase were used as recommended by the manufacturer (Promega). PCR amplifications were performed in a GeneAmp PCR system 2400 thermal cycler (Applied Science), using Expand high-fidelity DNA polymerase (Applied Science). Amplified DNA fragments were purified by using a PCR purification kit (Roche) and separated after digestion on 0.7% agarose gels. The digested DNA fragments were extracted from agarose gels with a centrifugal filter device (Montage DNA gel extraction kit; Millipore). Plasmids were first introduced by chemical transformation into E. coli DH5α and then into E. coli SCS110, and the resulted unmethylated plasmids were transferred into the B. cereus strains by electroporation, as described above. The expected sequence was confirmed by DNA sequencing for all constructions (GATC Biotech).

### Growth and fluorescence monitoring.

One purified colony of a 48-h culture was grown overnight at 30°C in mAOAC broth for each tested B. cereus strain. An aliquot of the suspension was inoculated at an initial *A*_600_ of 0.1 into 25 ml of mAOAC and incubated at 30°C under shaking at 200 rpm. This suspension was grown until the *A*_600_ was 0.5 and was then 10-fold diluted in mAOAC at pH 7.0 or pH 5.0, and 250 μl was dispensed into black-walled, clear-bottom 96-well microplates (Thermo Fisher Scientific). A spore inoculum containing 10^7^ CFU of spores/ml was inoculated into mAOAC broth in microplates, in the same way as described above for vegetative cells. The microplates were then incubated in a thermostatically controlled incubator at the tested temperature under shaking at 200 rpm. One hundred-microliter volumes were sampled at regular time points in dedicated wells to determine the changes in the CFU counts with time. The *A*_600_ and the fluorescence level of the cultures induced by the activity of the *cshA* or *abrB* promoter were measured in the other wells with a microplate reader (Infinite 200 Pro; Tecan). The excitation and emission wavelengths were 395 nm and 509 nm, respectively, for GFP and 587 nm and 610 nm, respectively, for the mCherry protein. The fluorescence of a sample was defined as the total fluorescence measured in the microplate wells minus the mean fluorescence of the noninoculated wells and was expressed in relative fluorescence units (RFU). CFU counts were obtained by plating serial dilutions on LB agar plates incubated overnight at 30°C. Each experiment was performed in triplicate with independently prepared bacterial cultures. At each sampling time, cell morphology and fluorescence were observed with an epifluorescence microscope (BX-61; Olympus) at a ×1,000 magnification. Images were taken with a digital camera (Orca Flash 4.0 LT; Hamamatsu).

### Determination of onsets of promoter activity, biomass increase, and CFU count increase.

To have a sufficient number of points to determine the results at the different times of interest, samples were taken every half hour or every hour throughout the lag phase period and at regular time intervals in the subsequent phases. At 12°C, experiments with different and complementary sampling times were necessary to cover the whole growth period. Changes in CFU counts, *A*_600_ values, and fluorescence values consisted of two parts: (i) first, they remained unchanged (horizontal lines), and (ii) then, they increased with time. The times to the onset promoter activity (revealed by the increase in the fluorescence [λ*_F_*]; the biomass increase, determined by an increase in the *A*_600_ [λ*_A_*]; and the increase in the number of CFU per milliliter [λ*_C_*]) were taken as the time of the intersection between these two parts and were estimated by minimizing the sum-of-squared error using the Microsoft Excel 2010 solver add-in program. To verify that the estimated values obtained did not deviate from the measured values, the root mean square error (RMSE) of all variables under all conditions was calculated (65). RMSE was normalized (NRMSE) to facilitate the comparison between our data sets with different scales. The formulas used to perform these calculations were the following:

$$\text{RMSE}=\sqrt{\frac{{\sum dt}^{2}}{T}}$$

and

$$\text{percent NRMSE}=\frac{\text{RMSE}}{yt_{\text{max}}-yt_{\text{min}}}\times 100$$

where *dt*^2^ is the square of the difference between the estimated value and the measured value at time *t*, *T* is the total number of estimations, and *yt*_max_ and *yt*_min_ correspond to the maximum and minimum estimated values, respectively.

The results of NRMSE are presented in Tables S1 to S4 in the supplemental material. The percent NRMSE was lower than 10% for >90% of the *A*_600_-versus-time curves (*n* = 88) and lower than 15% for >90% of the number of CFU per milliliter-versus-time curves (*n* = 87) and fluorescence-versus-time curves (*n* = 88).

### Measurement of stability of fluorescent proteins.

The stability of the GFP and mCherry fluorescence in the transformed cells was measured. All the strains were grown in mAOAC under shaking overnight. Cells were harvested, washed, and resuspended in fresh mAOAC medium to an OD at 600 nm of 1 in the presence of 200 μg/ml of chloramphenicol as a *de novo* protein synthesis inhibitor. Cells were incubated for 48 h at 30°C under shaking in an Infinite Pro200 apparatus (Tecan), and the fluorescence intensities were recorded every 15 min using the appropriate filters. Corrected fluorescence intensities (the fluorescence of the reporter strain minus the nonspecific fluorescence of the medium) were plotted as a function of time.

### Statistical analysis.

The results are expressed as the means from three independent biological replicates. A Student's *t* test was used to compare the mean values with the null hypothesis, which was rejected for *P* values of <0.05.

## Supplementary Material

Supplemental file 1
